# Puerperal Fever

**Published:** 1854-05

**Authors:** Philip S. Field

**Affiliations:** Baltimore Alms-House


### IOWA MEDICAL JOURNAL.

VOL. I. KEOKUK, IOWA, MAY, 1854. NO. 10.

### ORIGINAL COMMUNICATIONS.

### *A Case of Puerperal Fever.*

BY PHILIP S. FIELD, M. D.

Mary McDonald, aged 22, native of Ireland—but recently arrived in this country, was admitted to the Baltimore Alms-House, March 9th, 1854. Being near her confinement, she was immediately transferred to the lying-in-ward. She has been in Baltimore but one day, having arrived in this city after a tedious journey of several days from New York. She had been obliged to sleep out one night, having no money to obtain lodgings. When called to attend her, I found her in labor and by an examination, (the bag of waters having been previously ruptured) discovered the head in the first position. The pains were very severe, and continued so for nearly five hours. As the head had made no progress, I concluded with the approval of Dr. W., the visiting physician, to apply the forceps. After some exertion I succeeded in delivering her of a dead child.

The death of the child was doubtless caused by long continued pressure before the application of the forceps, as well as by the compression of the cord which was wrapped twice around the child's neck. After waiting nearly an hour and finding the pains insufficient, I administered ten grains of Ergot. In a few moments the uterus contracted firmly and the placenta was expelled without hemorrhage. I ordered,

℞ Hydrarg. Proto Chlor. grs. xij.
Pulv. Opii. grs. iss.
S To be given at once.

This I deemed necessary to counteract any inflammatory symptoms which might arise from the prolonged labor and the use of instruments. She continued tolerably well until the night of March 10th, when she complained of slight pain over the hypogastric region and was very restless all night. Ordered a large dose of Ol. Ricini.

March 11th. Half-past ten A. M. Suffering with a high fever. Pulse 140, and irritable. Respiration 52. Tongue dry and coated. Lochia has ceased. I bled her copiously and ordered

℞ Hydrarg. Chlor. Mit. grs. xij.
Pulv. Ipecac, grs. iv.
Pulv. Opii. grs. iss.
M. ft. pill viij. S One every hour and a half.

5 O'CLOCK, P. M. Pulse 142, very weak. Respiration as in the morning. Pain in the abdomen intense, with great tympanitis. Ordered, warm cataplasms and an injection of warm milk and water for the vagina—also,

℞ Ol. Terebinth, ℥ss.

Ol. Ricini, ℥i.

Muc. G. Acaciæ, ℥iv.

M. ft. Enema. S At once.

9 O'CLOCK, P. M. Pulse 150, irritable. Respiration 54. Complains of great thirst and the distension of her abdomen. Her appearance indicates intense suffering. As the first injection had failed to operate and the tympanitis was on the increase, I ordered

℞ Ol. Terebinth, ℥ss.

Tinct. Assafœtida, ℥iiss.

Muc. G. Acaciæ, ℥ij.

M. ft. Enema. S At once.

I also increased the dose of opium, giving half a grain at each dose.

12 O'CLOCK, P. M. Condition much the same. The pains are becoming intermittent in their character. During the intermissions, she has little or no pain.

March 12th. 7 o'clock, A. M. Tongue dry, not so much coated. Tympanitis less. Complains of little or no pain. Pulse 132. Respiration 36. Lochia not yet returned. Slept well after 12 o'clock. Continue the Calomel and Opium. Discontinue the Ipecac.

March 13th. 6 A. M. Has no pain. Tympanitis less. Countenance not so anxious. Pulse 140, very weak. Respiration 36. Ordered Brandy in small doses often repeated, with wine whey.

1,30 P. M. Pulse 150. Respiration as in the morning. The stomach is so irritable that nothing given by the mouth is retained. Extremities growing cold.

4,45 P. M. Pulse 120, scarcely appreciable. Respiration hurried. Extremities cold. Stomach still irritable. Has no pain. Intellect perfectly clear.

9 O'CLOCK, P. M. Pulse inappreciable. Pain in abdomen returning. Sinking very rapidly. Continue Brandy.

10,15 P. M. Excessive vomiting. Pain is very intense. Involuntary passage of fæces. Subsultus tendinum, somewhat delirious, has not slept.

10,45 P. M. Attempted to relieve the irritability of stomach by administering Hydrocyanic Acid, but without effect. From this time she sank rapidly and died at 4 A. M. Her intellect during the last hours of life was unclouded.

Autopsy eleven hours after death. Body very fat, face full.—There was slight rigidity of the upper extremities—legs rigid, ecchymosis of the posterior and depending portions of the body. Abdomen much distended. Opened by an incision through the *Linea Alba.* Walls of the abdomen very thick. Stomach and intestines much swollen from gas. The peritoneal intestinal convolutions were glued together by adhesions, roughened by lymph and much congested.—The peritoneal sac contained about two quarts of sero-purulent fluid, with flakes of lymph floating in it. Uterus about the size of a child's head. The walls near the fundus were an inch and a half in thickness and of a bluish color. On opening the organ, the attachment of the placenta was visible, the middle portion being of a gamboge yellow color—lower cervical portion gangrenous. The metro-peritoneal surface was uninflamed. The ovaries and their peritoneal covering were much congested. The disease seemed to be confined to the superficial lining membrane of the uterus, there being no evidence of any inflammation in the coats of the veins. The lower portion of the lining membrane of the uterus was gangrenous. The vagina presented a healthy appearance.

Baltimore Alms-House, March 30, 1854.

